# Increased risk of inflammatory bowel disease in ankylosing spondylitis compared to psoriasis

**DOI:** 10.3389/fimmu.2026.1762379

**Published:** 2026-02-06

**Authors:** Zhen He, Yi-Nan Zhang, James Cheng-Chung Wei, Sheng-Ming Dai

**Affiliations:** 1 Department of Rheumatology and Immunology, Shanghai Sixth People’s Hospital Affiliated to Shanghai Jiao Tong University School of Medicine, Shanghai, China; 2 Institute of Medicine, College of Medicine, Chung Shan Medical University, Taichung, Taiwan; 3 Department of Allergy, Immunology and Rheumatology, Chung Shan Medical University Hospital, Taichung, Taiwan; 4 Graduate Institute of Integrated Medicine, China Medical University, Taichung, Taiwan

**Keywords:** ankylosing spondylitis, epidemiology, inflammatory bowel disease, psoriatic disease, risk factor

## Abstract

**Background:**

While ankylosing spondylitis (AS) and psoriatic arthritis (PsA) share similar immune dysregulation, their relative risks for inflammatory bowel disease (IBD), including definitive subtypes (Crohn’s disease [CD] and ulcerative colitis [UC]) and possible subtypes (indeterminate colitis [IC] and microscopic colitis [MC]), remain unquantified. We aimed to establish comparative IBD risk gradients among AS, psoriasis (PSO), and PsA cohorts.

**Methods:**

The study utilized a long-term retrospective cohort design by analyzing an electronic health record database. Propensity score matching (PSM) was used to adjust multiple confounders. Cox proportional hazards models and log rank test were employed to evaluate the risk of IBD development.

**Results:**

The study included 26,610 patients with AS and 322,317 with PSO (2005–2023). After PSM, 26,569 matched pairs were analyzed. Compared to PSO, AS was associated with a significantly higher risk of definite IBD [hazard ratio (HR) = 2.96, 95% CI: 2.64–3.33], CD (HR = 3.38, 95% CI: 2.90–3.94), UC (HR = 2.43, 95% CI: 2.07–2.85), and IC (HR = 2.45, 95% CI: 1.33–4.51), but not MC. Subgroup analyses confirmed a consistently higher IBD risk in AS across all ages, sexes, races, BMI categories, and comorbidity profiles. Compared to the general population, AS conferred the highest independent risk for definite IBD (HR = 4.22, 95% CI: 3.60–4.94), followed by PsA (HR = 1.52) and PSO (HR = 1.37). AS also showed a 2.60-fold higher definite IBD risk than PsA (95% CI: 2.32–2.92).

**Conclusions:**

AS is the phenotype most strongly associated with IBD among the studied spectrum. Compared to PSO, AS confers a significantly higher risk of CD, UC, and IC, and it carries a greater burden of definite IBD than PsA or the general population.

## 1 Introduction

Spondyloarthritis (SpA) encompasses a spectrum of chronic inflammatory disorders targeting the axial skeleton and peripheral joints, with systemic manifestations frequently involving the skin, eyes, and gastrointestinal tract. Key subtypes include ankylosing spondylitis (AS), reactive arthritis, inflammatory bowel disease (IBD)-associated arthritis, and psoriatic arthritis (PsA) (1). These subtypes share common immunogenetic pathways (2), such as the dysregulation of the interleukin (IL)-23/T helper 17 (Th17) axis (3–6). Notably, AS and psoriatic disease exhibit distinct tissue tropisms. AS is characterized by axial skeletal damage, while psoriatic disease is mainly manifested as cutaneous and peripheral joint inflammation. Both conditions are associated with a high prevalence of extra-musculoskeletal and peripheral manifestations, particularly IBD (7–10).

IBD comprises a wide range of chronic immune-mediated intestinal disorders, which have traditionally been classified into Crohn’s disease (CD) and ulcerative colitis (UC) (11). Epidemiological studies confirm an elevated IBD risk in patients with AS, PsA, or psoriasis (PSO) compared to the general population (12–20). Emerging data collectively suggest that this risk may follow a gradient across these related phenotypes—for instance, some studies comparing AS and PsA suggest a higher risk in AS, while analyses within the psoriatic disease spectrum indicate that risk increases from PSO to PsA (13, 14, 21–24). However, these evidence streams have not been integrated within a single, methodologically consistent framework. Thus, while a risk hierarchy is biologically plausible, a unified analysis providing directly comparable risk estimates for AS, PSO, and PsA is scarce.

Beyond CD and UC, emerging entities like indeterminate colitis (IC) and microscopic colitis (MC) challenge traditional classification (25–27). IC, a provisional diagnosis with overlapping CD/UC features, may represent a transitional gut inflammation state (28). To date, however, epidemiological and mechanistic studies investigating the association between IC and other immune−mediated diseases remain notably scarce. MC, an immune-mediated disorder linked to autoimmunity (29, 30), has reported associations with both PSO and AS (31, 32), but its comparative risk across these cohorts is undefined.

Furthermore, meaningful interpretation of such comparative studies is complicated by significant heterogeneity in environmental exposures (e.g., smoking) (33), therapeutic interventions (e.g., TNF inhibitors) (34), and ethnic factors, all of which can obscure disease-specific risk estimates (8). Consequently, there is a lack of comprehensive studies that simultaneously directly compare the risk of definitive IBD (CD, UC) and possible IBD (IC, MC) across AS, PSO, and PsA and rigorously adjust for this broad range of potential confounders.

To address these gaps, we conducted a large-scale, propensity score-matched (PSM) cohort study. Our primary objectives were (1) to perform a direct, adjusted comparison of overall IBD and subtype risk between AS and PSO patients and (2) to establish and compare the independent IBD risk associated with AS, PSO, and PsA by deriving standardized hazard ratios (HR) relative to matched general population cohorts. This approach aims to delineate a risk gradient to inform tailored clinical surveillance.

## 2 Methods

### 2.1 Data sources

The database was from a collaborative electronic health record database (https://trinetx.com), a federated health program which provides real-time updates of data from electronic healthcare records, including demographics, vital statuses, laboratory results, diagnoses, and treatments (35). The personal identification is protected. The International Classification of Diseases, Tenth Revision, Clinical Modification (ICD-10-CM) codes were used to classify diseases. In addition, the study was approved under the authority of the Institutional Review Board of Chung Shan Medical University Hospital (no.: CS2-21176).

### 2.2 Study design and patient selection

We select patients aged 20 or older from the database who had been diagnosed with PSO, PSA, and AS in accordance with the ICD-10-CM code during the period from January 1, 2005 to December 31, 2023 (**Supplementary Table S1**). The AS cohort, defined by ICD-10 codes, primarily represents patients with radiographic axial spondyloarthritis (axSpA). In these cohorts, patients diagnosed with IBD were designated as the primary outcome of this study. Those patients who had been diagnosed with IBD prior to the index date were excluded from the analysis. Some covariates, namely, age, sex, body mass index (BMI), race, medical utilization, comorbidities, and medication usage, were analyzed (**Supplementary Table S2**). To mitigate the impact of confounding factors, we employed the PSM method with a 1:1 ratio matching based on the aforementioned covariates. All covariates listed in **Supplementary Table S2** were included in the PSM model. Comparisons between the AS and PSO cohorts both before and after matching were investigated using a standardized mean difference (SMD). It was considered as well matched if the SMD was lower than 0.1. Subgroup analyses were further carried out to examine whether the risk of IBD in PSO and AS patients varied according to each covariate.

### 2.3 Statistical analysis

The baseline characteristics of the participants were expressed as numbers and percentages for categorical variables and means ± standard deviations for continuous variables. Chi-square and Student’s *t*-tests were applied to examine the categorical and continuous variables between the two cohorts. A Cox proportional hazards survival model was used to compare event rates over time between diagnostic groups. The HR and 95% confidence interval (CI) were calculated using univariate Cox proportional hazard model. The results were graphically inspected via Kaplan–Meier plots to validate the model assumptions. Log-rank tests were done to assess whether the survival curves differed between different cohorts.

## 3 Results

### 3.1 Comparison of baseline characteristics in AS and PSO patients

**Figure 1:**
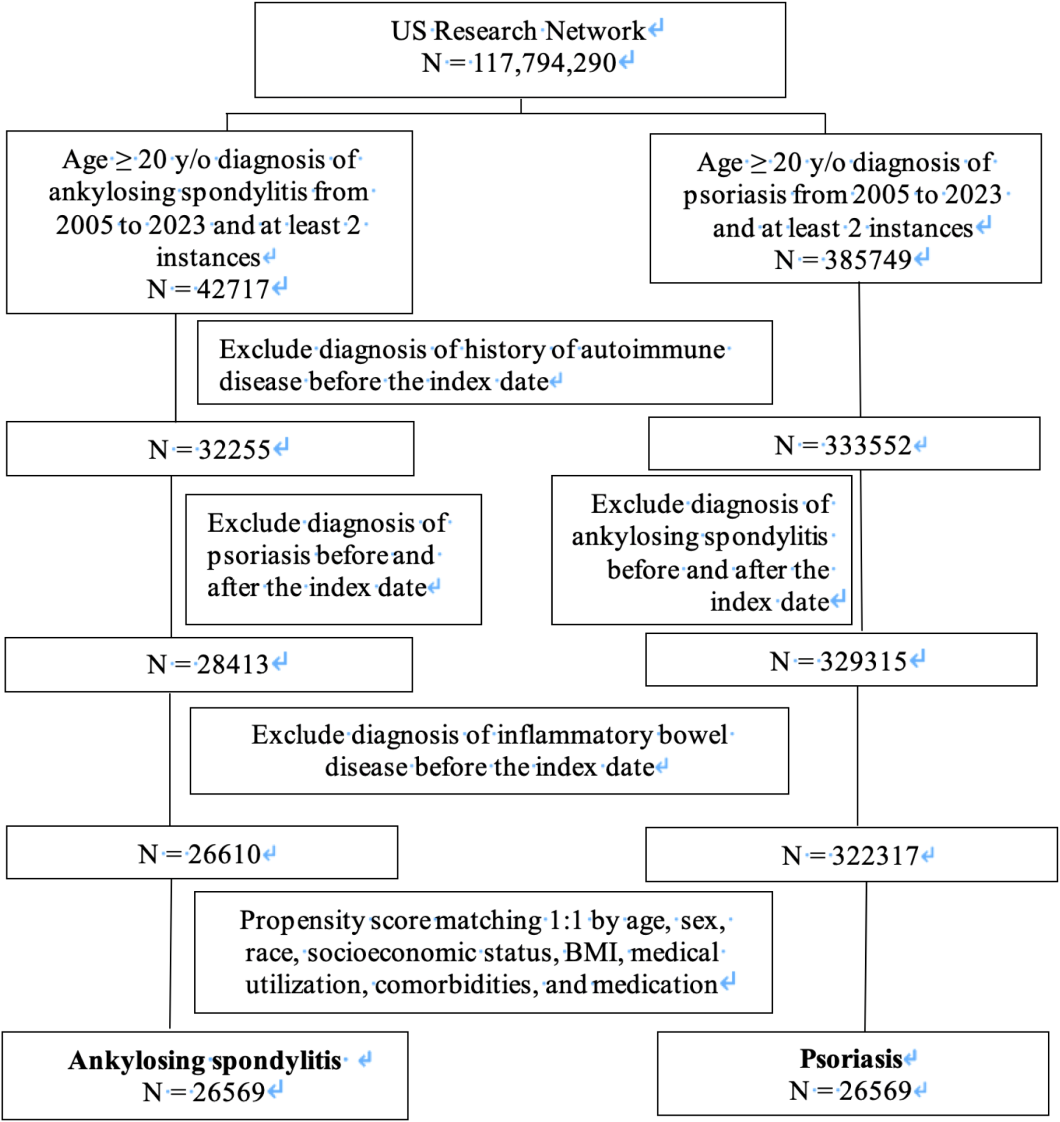
Flow chart of subject selection.

During the study period within the database, a total of 42,717 adults with AS and 385,749 adults with PSO, all aged 20 or older, were identified. Subsequent to the application of exclusion criteria and the removal of patients diagnosed with IBD, 26,610 individuals with AS and 322,317 individuals with PSO were singled out (**Figure 1**). The baseline characteristics, including age, sex, race, social economic status, BMI, medical utilization, comorbidities, and medication intake, were compared between the AS and PSO groups (**Supplementary Table S2**). Before propensity matching, the AS group was mainly composed of male patients (58.60%) and was marginally younger than the PSO group (49.76 ± 16.39 years versus 51.44 ± 15.70 years). The AS group had a relatively higher proportion of Asian, Black or African Americans, and American Indian or Alaska native compared to the PSO group. PSO patients exhibited higher rates of comorbidities, such as hypertensive diseases (18.87% versus 15.36%), dyslipidemia (15.85% versus 12.36%), diabetes mellitus (9.56% versus 6.79%), liver diseases (3.29% versus 2.19%), cerebrovascular diseases (2.51% versus 2%), and nicotine dependence (5.34% versus 3.62%). In terms of medical usage, a greater number of PSO patients were treated with corticosteroids (21.23% versus 19.86%) and cyclosporine (0.36% versus 0.26%), while AS patients tended to receive methotrexate (1.75% versus 1.44%). Regarding the application of anti-tumor necrosis factor (TNF) antibodies and anti-interleukin (IL)-17 antibodies, more AS patients received etanercept, infliximab, adalimumab, golimumab, certolizumab Pegol, and secukinumab (**Supplementary Table S2**). After PSM, 26,569 patients were selected for each group, with all baseline features well balanced (**Supplementary Table S2**).

### 3.2 Overall risk of IBD in AS compared to PSO

**Figure 2:**
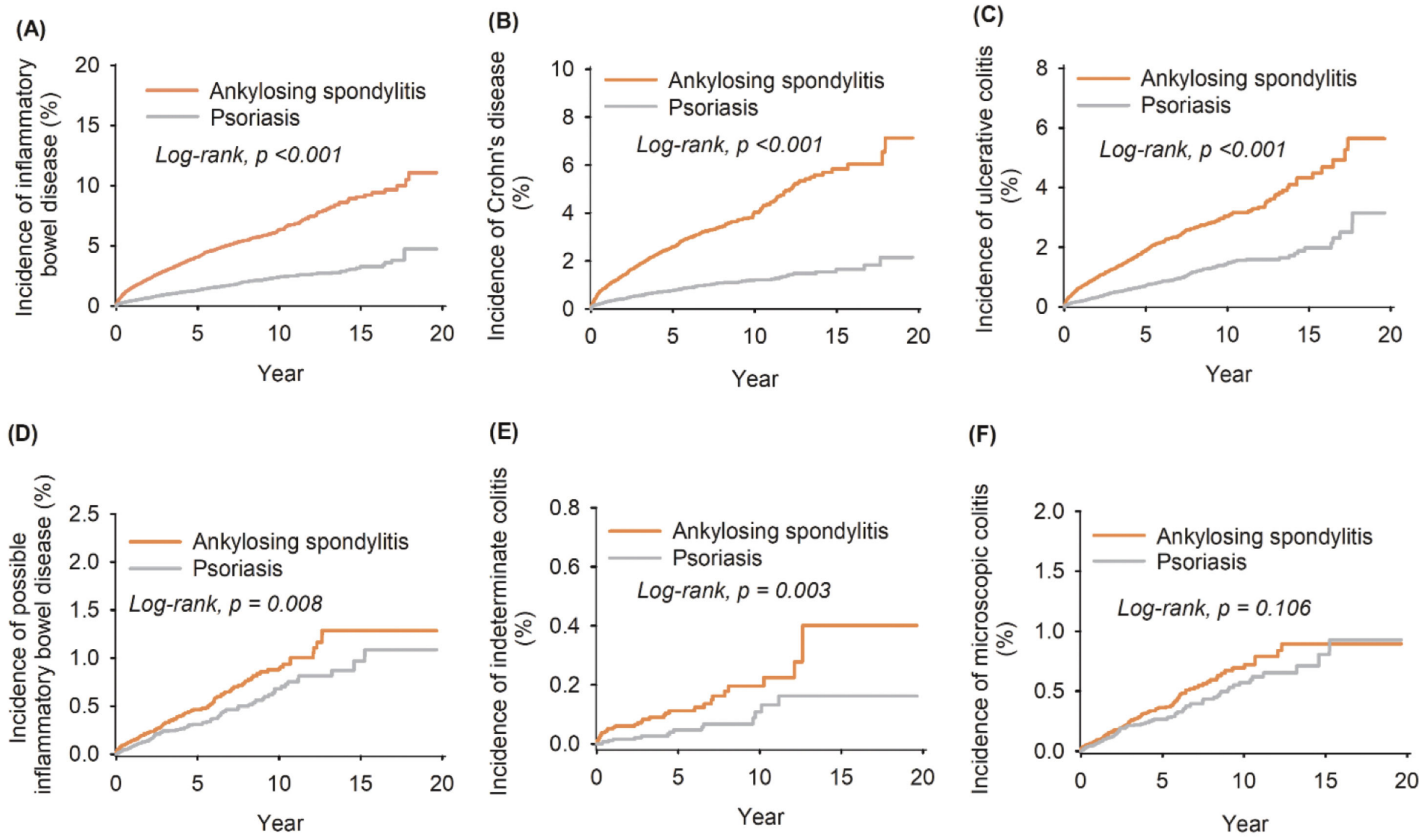
Kaplan–Meier plots and log-rank tests for risk of inflammatory bowel disease exposed to ankylosing spondylitis compared to psoriasis. **(A–F)** Separately indicated definite inflammatory bowel disease, Crohn’s disease, ulcerative colitis, possible inflammatory bowel disease, indeterminate colitis, and microscopic colitis. The orange straight line denoted ankylosing spondylitis, while the gray straight line indicated psoriasis. *P* < 0.05 was considered statistically significant.

Following PSM analysis based on age, sex, race, social economic status, BMI, medical utilization, comorbidities, and medication intake, the risk of definite and possible IBD in AS and PSO patients was evaluated. Herein CD and UC were defined as definite IBD, while IC and MC were defined as possible IBD. The cumulative incidence of CD, UC, and IC was higher in the AS group than in the PSO group (7.13% versus 2.15% for CD, 5.64% versus 3.14% for UC, and 0.4% versus 0.16% for IC), whereas no difference was observed in the incidence rate of MC between the two groups (0.9% versus 0.93%). AS was associated with a substantially higher risk of definite IBD in comparison to PSO, with a HR of 2.96 (95% CI: 2.64–3.33; log-rank test, *P* < 0.001) (**Table 1**, **Figure 2**). For possible IBD, patients with AS had a stronger association with IC (HR = 2.45, 95% CI: 1.33–4.51; log-rank test, *P* = 0.003) (**Table 1**, **Figure 2**), but not for MC (HR = 1.27, 95% CI: 0.95–1.70; log rank test, *P* = 0.106) (**Table 1**, **Figure 2**). Sensitivity analysis across different network databases corroborated these results (**Supplementary Table S3**).

**Table 1:** Risk of inflammatory bowel disease exposed to ankylosing spondylitis compared to psoriasis.

Cohort	No. of events (*N* = 26,569)			Cumulative incidence (%)	
	AS	PSO	HR (95% CI)	AS	PSO
Definite IBD	1,030	389	2.96 (2.64–3.33)^a^	11.08	4.75
Crohn’s disease	655	215	3.38 (2.90–3.94)^a^	7.13	2.15
Ulcerative colitis	476	219	2.43 (2.07–2.85)^a^	5.64	3.14
Possible IBD	129	101	1.42 (1.10–1.85)^a^	1.29	1.09
Indeterminate colitis	33	15	2.45 (1.33–4.51)^a^	0.40	0.16
Microscopic colitis	98	86	1.27 (0.95–1.70)	0.90	0.93

AS, ankylosing spondylitis; PSO, psoriasis; IBD, inflammatory bowel disease; CI, confidence interval.

a Considered statistically significant.

### 3.3 Subgroup analysis of IBD risk in AS compared to PSO

To further illustrate the elevated risk of IBD associated with AS in contrast to PSO, we carried out subgroup analysis based on age, sex, BMI, race, and comorbidities (**Table 2**). In general, among the 20–40, 41–64, and ≥65 age groups, the presence of IBD patients was observed in 349/7,873 (4.43%), 438/11,078 (3.95%), and 168/5675 (5.92%) of AS patients, respectively, while in the PSO population, the corresponding figures were 109/7873 (1.38%), 143/11,078 (1.29%), and 72/5675 (1.27%). When stratified by gender, the incident rates of IBD were persistently higher in AS patients regardless of gender (**Table 2**). Additionally, differences in IBD incidence were consistently detected between the AS and PSO groups across diverse BMI categories (**Table 2**). With regard to race, White patients with AS (HR = 3.01, 95% CI: 2.64–3.44), Black or African American patients with AS (HR = 5.48, 95% CI: 3.03–9.94), and Asian patients with AS (HR = 2.14, 95% CI: 1.03–4.43) exhibited a higher susceptibility to developing IBD when compared to their counterparts with PSO. Moreover, the risk of IBD in AS patients remained higher than that in PSO patients, irrespective of whether they were complicated by hypertensive diseases (HR = 1.75, 95% CI: 1.29–2.37) and dyslipidemia (HR = 1.59, 95% CI: 1.12–2.23) (**Table 2**).

**Table 2:** Subgroup analysis of risk of inflammatory bowel disease exposed to ankylosing spondylitis compared to psoriasis.

Variable	Ankylosing spondylitis		Psoriasis		
	*N*	No. of events	*N*	No. of events	HR (95% CI)
Age, years					
20–40	7,873	349	7,873	109	3.53 (2.84–4.37)^a^
41–64	11,078	438	11,078	143	3.34 (2.77–4.04)^a^
≥65	5,675	168	5,675	72	2.73 (2.07–3.60)^a^
Sex					
Female	9,265	341	9,265	149	2.58 (2.13–3.13)^a^
Male	15,157	620	15,157	197	3.47 (2.95–4.07)^a^
Race					
White	19,572	795	19,572	295	3.01 (2.64–3.44)^a^
Black or African American	1,590	67	1,590	13	5.48 (3.03–9.94)^a^
Asian	951	21	951	11	2.14 (1.03–4.43)^a^
BMI (kg/m^2^)					
<30	4,588	172	4,588	85	2.30 (1.77–2.98)^a^
≥30	2,582	73	2,582	37	2.34 (1.57–3.48)^a^
Comorbidities					
Hypertensive diseases	3,965	106	3,965	71	1.75 (1.29–2.37)^a^
Dyslipidemia	3,180	77	3,180	57	1.59 (1.12–2.23)^a^
Cerebrovascular diseases	519	11	519	12	1.12 (0.49–2.54)

If the patient’s count is 1–10, the results indicate a count of 10.

CI, confidence interval; BMI, body mass index.

a Considered statistically significant.

### 3.4 Analysis of IBD risk among different groups

Subsequently, we assessed the risk of IBD among different groups. We discovered that in the general population, the cumulative incidence of definite IBD ranged from 2.18% to 2.85%. In contrast, it was approximately 11% in patients with AS, 4.08% in those with PSO, and around 5% in patients with PsA, respectively (**Table 3**). A continuously elevated risk of developing IBD was observed in patients with AS (HR = 4.22, 95% CI: 3.6–4.94), PSO (HR = 1.37, 95% CI: 1.31–1.45), and PsA (HR = 1.52, 95% CI: 1.36–1.70) in comparison to the general population. Further comparison with PsA showed that AS was associated with a considerably higher risk of definite IBD, with a HR of 2.60 (95% CI: 2.32–2.92) (**Table 3**).

**Table 3:** Risk of inflammatory bowel disease among different groups.

Group	*N*	No. of events	HR (95% CI)	Cumulative incidence (%)
Group				
Psoriatic arthritis	25,377	408	Reference	5.52
Ankylosing spondylitis	25,377	989	2.60 (2.32–2.92)^a^	11.14
Group				
General population	24,276	187	Reference	2.18
Ankylosing spondylitis	24,276	948	4.22 (3.61–4.94)^a^	10.68
Group				
General population	306,769	2,301	Reference	2.85
Psoriasis	306,769	4,265	1.37 (1.31–1.45)^a^	4.08
Group				
General population	59,741	484	Reference	2.45
Psoriatic arthritis	59,741	943	1.52 (1.36–1.70)^a^	5.16

The general population was identified by ICD-10-CM = Z02 (encounter for administrative examination), excluding ICD-10-CM = M45, M46, and L40.

CI, confidence interval.

a Considered statistically significant.

## 4 Discussion

In this large-scale, propensity score-matched cohort study using longitudinal real-world data, we provide a comprehensive, direct comparison of IBD risk between patients with AS and those with psoriatic disease (PSO/PsA), integrating both definitive (CD and UC) and possible (IC and MC) subtypes. Our analysis reveals a clear and consistent risk gradient: AS conferred the highest independent risk for definite IBD compared to the general population (HR = 4.22), followed by PsA (HR = 1.52), with PSO alone showing a more modest elevation (HR = 1.37). Critically, the risk of definite IBD in AS was nearly threefold higher than in PSO (HR = 2.96) and 2.6-fold higher than in PsA (HR = 2.60). We further identified a novel, specific association between AS and an increased incidence of IC, whereas the risk of MC was comparable between AS and PSO.

Our findings provide independent validation and quantitative refinement to the risk hierarchy suggested in prior literature. Consistent with a large Swedish national register study, which reported incidence rate ratios of 6.2 for IBD in AS versus 2.3 in PsA (22), we observed a markedly higher risk burden in AS compared to PsA (HR = 4.22 vs. 1.52). The direct, adjusted comparison between AS and PSO in our study (HR = 2.96) provides crucial missing data that refines this gradient. Prior studies within the psoriatic disease spectrum have detailed increased IBD risk in PSO (13, 14), further amplified by concomitant PsA (21), but often lacked a methodologically parallel AS cohort for direct comparison. Our study addresses this gap by analyzing all three cohorts within the same framework, employing rigorous propensity score matching to balance key demographic, comorbidity, and treatment confounders.

Our analysis also included an evaluation of possible IBD subtypes. To our knowledge, direct comparisons of IC and MC risks between AS and PSO in large cohorts have been limited. In our cohort, AS was associated with a significantly higher risk of IC compared to PSO (HR = 2.45), a difference that has not been well characterized in prior large-scale studies. IC is a recognized diagnostic category reserved for chronic colitis that cannot be definitively classified as either CD or UC based on endoscopic, histological, and radiographic features (36). It often represents a transitional or overlapping inflammatory state within the IBD spectrum (28). Our data, linking AS specifically to a higher incidence of IC, suggest that the gut inflammation in AS may present in an atypical or evolving pattern that defies early conventional classification. In contrast, the risk of MC was found to be comparable between AS and PSO patients. MC is a distinct, immune-mediated colonic disorder characterized by chronic watery diarrhea and specific histopathological findings (lymphocytic or collagenous colitis) (37–39). Its pathogenesis involves mucosal dysregulation of T-cell responses (40) and is known to share genetic susceptibility loci (41) and immunological pathways with other autoimmune conditions, notably psoriasis (31). Our observation of comparable MC risk between AS and PSO is supported by large-scale epidemiological evidence. A nationwide case–control study confirmed that MC is significantly associated with a wide range of autoimmune diseases, demonstrating an increased odds ratio for both AS and psoriasis (32). However, the precise mechanisms underlying MC, and its specific associations with AS and PSO, remain to be fully elucidated.

Some factors are significant influencers in the development of IBD (42–44). Uria Shani et al. carried out a population-based study with the aim of exploring the predictors associated with the development of IBD among patients with PSO. They determined that advanced age, male gender, and Jewish ethnicity were risk factors for IBD (14). Therefore, in order to investigate whether the risk factors for IBD had an impact on the incident rate of IBD between AS and PSO, we performed a subgroup analysis based on several cofounding variables. We discovered that the relatively higher risk of IBD in patients with AS compared to those with PSO was consistently observed in different subgroups categorized by age, sex, BMI, and race. Our results, at least to some extent, indicated the robust and enhanced effect of AS on the risk of IBD in comparison to PSO.

The marked risk gradient identified necessitates a re-evaluation of clinical surveillance. For patients with AS, the substantially elevated risks for CD, UC, and specifically IC warrant a high index of suspicion. Proactive inquiry about gastrointestinal symptoms should be integrated into rheumatological follow-up, with prompt gastroenterological referral for symptomatic patients. The specific increase in IC risk suggests that gut inflammation in AS may often be atypical, making comprehensive ileocolonoscopy with serial biopsies crucial for accurate classification.

Some limitations within the study warrant acknowledgment. First, despite the independent analyses for patients with AS, PsA, and PSO, a certain degree of overlap among the diagnoses remain unavoidable. Second, patients with a milder disease who were managed in primary healthcare might be overlooked, thereby potentially limiting the generalizability of the current findings. Third, the observational design precludes confirmation of causality, and residual confounding from unmeasured factors (e.g., smoking intensity, disease duration, and time-varying treatment exposure) cannot be excluded. Fourth, our AS cohort, defined by ICD-10 codes, primarily captures patients with axSpA. Therefore, our findings should be interpreted in the context of established AS and may not be generalizable to the broader spectrum of non-radiographic axSpA without further validation.

In conclusion, this study delineates a clear gradient of IBD risk across AS, PsA, and PSO, with AS representing the highest-risk phenotype. The specifically elevated risk of IC in AS underscores the importance of heightened clinical suspicion and tailored diagnostic evaluation in this population. These findings together support the recognition of AS as a distinct high-risk IBD phenotype, suggesting that surveillance and management strategies should be differentiated from those for PSO and PsA.

## Data Availability

The original contributions presented in the study are included in the article/**Supplementary Material**. Further inquiries can be directed to the corresponding authors.
