# Supplementary material for: Increased risk of inflammatory bowel disease in ankylosing spondylitis compared to psoriasis
Source: Front Immunol. 2026 Feb 6;17:1762379. doi: 10.3389/fimmu.2026.1762379 (PMC12920575; doi:10.3389/fimmu.2026.1762379)
Supplement: Supplementary file 1 [file DataSheet1.docx]

Table S1. Covariates and the corresponding codes used in our study

| Covariates | Code |
| --- | --- |
| **Social econo****mic status** |  |
| Persons with potential health hazards related to socioeconomic and  psychosocial circumstances | ICD-10-CM = Z55-Z65 |
| Problems related to housing and economic circumstances | ICD-10-CM = Z59 |
| Problems related to education and literacy | ICD-10-CM = Z55 |
| Problems related to employment and unemployment | ICD-10-CM = Z56 |
| Occupational exposure to risk factors | ICD-10-CM = Z57 |
| **Comorbidities** |  |
| Nicotine dependence | ICD-10-CM = F17 |
| Alcohol related disorders | ICD-10-CM = F10 |
| Hypertensive diseases | ICD-10-CM = I10-I1A |
| Disorders of lipoprotein metabolism and other dyslipidemias | ICD-10-CM = E78 |
| Diabetes mellitus | ICD-10-CM = E08-E13 |
| Depressive episode | ICD-10-CM = F32 |
| Ischemic heart diseases | ICD-10-CM = I20-I25 |
| Diseases of liver | ICD-10-CM = K70-K77 |
| Cerebrovascular diseases | ICD-10-CM = I60-I69 |
| **Medications** |  |
| Hormones/synthetics/modifiers | VA: HS000 |
| Antirheumatics | VA: MS100 |
| Corticosteroids for systemic use | ATC: H02 |
| Methotrexate | RxNorm: 6851 |
| Cyclosporine | RxNorm: 3008 |
| Ustekinumab | RxNorm: 847083 |
| **Anti-TNF antibody** |  |
| Etanercept | RxNorm: 214555 |
| Infliximab | RxNorm: 191831 |
| Adalimumab | RxNorm: 327361 |
| Golimumab | RxNorm: 819300 |
| Certolizumab pegol | RxNorm: 709271 |
| **Anti-IL17 antibody** |  |
| Secukinumab | RxNorm: 1599788 |
| Ixekizumab | RxNorm: 1745099 |
| Brodalumab | RxNorm: 1872251 |
| Bimekizumab | RxNorm: 2668041 |

ICD-10-CM: International Classification of Diseases, Tenth Revision, Clinical Modification; VA: Veterans Affairs Drug Classification system; ATC: Anatomical Therapeutic Chemical; RxNorm: Medical prescription normalized Medical prescription. TNF, tumor necrosis factor; IL-17, interleukin-17.

Table S2. Demographic characteristics of ankylosing spondylitis and psoriasis

|  | Before PSM | |  |  | After PSM | |  |  |
| --- | --- | --- | --- | --- | --- | --- | --- | --- |
|  | Ankylosing spondylitis N = 26,610 | Psoriasis N = 322,317 | p | SMD | Ankylosing spondylitis N = 26,569 | Psoriasis N = 26,569 | p | SMD |
| **Age, Mean ± SD** | 49.67 ± 16.39 | 51.44 ± 15.70 | <0.001 | 0.110 | 49.70 ± 16.38 | 49.51 ± 16.00 | 0.179 | 0.012 |
| **Sex****, *n* (%)** |  |  |  |  |  |  |  |  |
| Female | 9949 (37.39) | 163,252 (50.65) | <0.001 | 0.270 | 9938 (37.40) | 10,093 (37.99) | 0.165 | 0.012 |
| Male | 15,592 (58.60) | 144,324 (44.78) | <0.001 | 0.279 | 15,562 (58.57) | 15,435 (58.09) | 0.264 | 0.010 |
| **Race****,** ***n* (%)** |  |  |  |  |  |  |  |  |
| White | 19,597 (73.65) | 244,207 (75.77) | <0.001 | 0.049 | 19,572 (73.67) | 19,841 (74.68) | 0.008 | 0.023 |
| Black or African American | 1708 (6.42) | 16,191 (5.02) | <0.001 | 0.060 | 1703 (6.41) | 1606 (6.05) | 0.082 | 0.015 |
| Asian | 979 (3.68) | 10,338 (3.21) | <0.001 | 0.026 | 976 (3.67) | 1018 (3.83) | 0.338 | 0.008 |
| American Indian or Alaska Native | 172 (0.65) | 1204 (0.37) | <0.001 | 0.038 | 171 (0.64) | 161 (0.61) | 0.582 | 0.005 |
| Native Hawaiian or Other Pacific Islander | 153 (0.58) | 2539 (0.79) | <0.001 | 0.026 | 153 (0.58) | 151 (0.57) | 0.908 | 0.001 |
| Other Race | 962 (3.62) | 9385 (2.91) | <0.001 | 0.040 | 958 (3.61) | 887 (3.34) | 0.092 | 0.015 |
| Unknown Race | 3039 (11.42) | 38,453 (11.93) | 0.014 | 0.016 | 3036 (11.43) | 2905 (10.93) | 0.071 | 0.016 |
| **Social economic status, *n* (%)** |  |  |  |  |  |  |  |  |
| Socioeconomic and psychosocial circumstances problem | 240 (0.90) | 8412 (2.61) | <0.001 | 0.130 | 239 (0.90) | 217 (0.82) | 0.301 | 0.009 |
| Housing/economic circumstances problem | 120 (0.45) | 5598 (1.74) | <0.001 | 0.124 | 119 (0.45) | 111 (0.42) | 0.597 | 0.005 |
| Problems related to education and literacy | 10 (0.04) | 112 (0.04) | 0.812 | 0.001 | 10 (0.04) | 10 (0.04) | 1.000 | <0.001 |
| Problems related to employment and unemployment | 26 (0.10) | 943 (0.29) | <0.001 | 0.044 | 26 (0.10) | 25 (0.09) | 0.889 | 0.001 |
| Occupational exposure to risk factors | 10 (0.04) | 141 (0.04) | 0.642 | 0.003 | 10 (0.04) | 10 (0.04) | 1.000 | <0.001 |
| **Medical utilization** |  |  |  |  |  |  |  |  |
| Ambulatory | 16,078 (60.42) | 195,594 (60.68) | 0.399 | 0.005 | 16,043 (60.38) | 15,950 (60.03) | 0.410 | 0.007 |
| Emergency | 2728 (10.25) | 28,568 (8.86) | <0.001 | 0.047 | 2719 (10.23) | 2580 (9.71) | 0.044 | 0.017 |
| Inpatient Encounter | 1644 (6.18) | 24,025 (7.45) | <0.001 | 0.051 | 1642 (6.18) | 1471 (5.54) | 0.002 | 0.027 |
| **BMI (Kg/m^2^)** |  |  |  |  |  |  |  |  |
| <30 | 4950 (18.60) | 54,837 (17.01) | <0.001 | 0.042 | 4935 (18.57) | 4788 (18.02) | 0.099 | 0.014 |
| ≥30 | 3382 (12.71) | 49,358 (15.31) | <0.001 | 0.075 | 3376 (12.71) | 3133 (11.79) | 0.001 | 0.028 |
| Mean ± SD | 29.44 ± 7.16 | 30.72 ± 7.64 | <0.001 | 0.173 | 29.45 ± 7.16 | 29.70 ± 7.21 | 0.028 | 0.036 |
| **Comorbidities** |  |  |  |  |  |  |  |  |
| Nicotine dependence | 962 (3.62) | 17,196 (5.34) | <0.001 | 0.083 | 960 (3.61) | 851 (3.20) | 0.009 | 0.023 |
| Alcohol related disorders | 326 (1.23) | 5676 (1.76) | <0.001 | 0.044 | 326 (1.23) | 279 (1.05) | 0.055 | 0.017 |
| Hypertensive diseases | 4087 (15.36) | 60,831 (18.87) | <0.001 | 0.093 | 4085 (15.38) | 3798 (14.30) | <0.001 | 0.030 |
| Dyslipidemia | 3290 (12.36) | 51,089 (15.85) | <0.001 | 0.100 | 3288 (12.38) | 3016 (11.35) | <0.001 | 0.032 |
| Diabetes mellitus | 1806 (6.79) | 30,808 (9.56) | <0.001 | 0.101 | 1804 (6.79) | 1566 (5.89) | <0.001 | 0.037 |
| Depressive episode | 1286 (4.83) | 19,746 (6.13) | <0.001 | 0.057 | 1280 (4.82) | 1129 (4.25) | 0.002 | 0.027 |
| Ischemic heart diseases | 1217 (4.57) | 15,854 (4.92) | 0.012 | 0.016 | 1217 (4.58) | 1108 (4.17) | 0.021 | 0.020 |
| Diseases of liver | 582 (2.19) | 10,602 (3.29) | <0.001 | 0.068 | 581 (2.19) | 501 (1.89) | 0.014 | 0.021 |
| Cerebrovascular diseases | 533 (2.00) | 8098 (2.51) | <0.001 | 0.034 | 533 (2.01) | 441 (1.66) | 0.003 | 0.026 |
| **Medications** |  |  |  |  |  |  |  |  |
| Hormones/synthetics/modifiers | 7456 (28.02) | 97,511 (30.25) | <0.001 | 0.049 | 7439 (28.00) | 7137 (26.86) | 0.003 | 0.025 |
| Antirheumatics | 6854 (25.76) | 49,702 (15.42) | <0.001 | 0.258 | 6813 (25.64) | 6686 (25.17) | 0.206 | 0.011 |
| Corticosteroids for systemic use | 5284 (19.86) | 6,8434 (21.23) | <0.001 | 0.034 | 5271 (19.84) | 5136 (19.33) | 0.140 | 0.013 |
| Methotrexate | 466 (1.75) | 4648 (1.44) | <0.001 | 0.025 | 460 (1.73) | 453 (1.71) | 0.815 | 0.002 |
| Cyclosporine | 69 (0.26) | 1148 (0.36) | 0.010 | 0.017 | 69 (0.26) | 61 (0.23) | 0.482 | 0.006 |
| **Anti-TNF antibody** |  |  |  |  |  |  |  |  |
| Etanercept | 576 (2.17) | 1727 (0.54) | <0.001 | 0.141 | 568 (2.14) | 565 (2.13) | 0.928 | 0.001 |
| Infliximab | 135 (0.51) | 486 (0.15) | <0.001 | 0.062 | 127 (0.48) | 128 (0.48) | 0.950 | 0.001 |
| Adalimumab | 1102 (4.14) | 3068 (0.95) | <0.001 | 0.204 | 1080 (4.07) | 1101 (4.14) | 0.646 | 0.004 |
| Golimumab | 103 (0.39) | 117 (0.04) | <0.001 | 0.076 | 80 (0.30) | 67 (0.25) | 0.283 | 0.009 |
| Certolizumab Pegol | 93 (0.35) | 148 (0.05) | <0.001 | 0.068 | 80 (0.30) | 68 (0.26) | 0.323 | 0.009 |
| **Anti-IL17 antibody** |  |  |  |  |  |  |  |  |
| Secukinumab | 131 (0.49) | 783 (0.24) | <0.001 | 0.041 | 130 (0.49) | 117 (0.44) | 0.407 | 0.007 |
| Ixekizumab | 29 (0.11) | 501 (0.16) | 0.061 | 0.013 | 29 (0.11) | 25 (0.09) | 0.586 | 0.005 |
| Brodalumab | 0 (0.00) | 15 (0.01) | 0.266 | 0.010 | 0 (0.00) | 0 (0.00) | <0.001 | <0.001 |
| Bimekizumab | 0 (0.00) | 0 (0.00) | <0.001 | <0.001 | 0 (0.00) | 0 (0.00) | <0.001 | <0.001 |

Quantitative data are expressed as mean ± standard deviation numerical data are expressed as number (%) if not stated otherwise, propensity score analysis was used to match demographic variables, comorbidities, and medications. A SMD value < 0.1 is considered well-matched. If the patient's count is 1-10, the results indicate a count of 10. BMI, body mass index; PSM, propensity score matching; SMD, standardized mean difference; TNF, tumor necrosis factor; IL-17, interleukin.

Table S3. Sensitivity analysis for risk of inflammatory bowel disease by different network databases

|  | Ankylosing spondylitis | | Psoriasis | |  |
| --- | --- | --- | --- | --- | --- |
|  | N | No. of event | N | No. of event | HR (95% CI) |
| EMEA network | 4222 | 151 | 4222 | 51 | 2.93 (2.13-4.02)^a^ |
| APAC network | 6792 | 37 | 6792 | 12 | 2.74 (1.42-5.25)^a^ |

EMEA (Europe, Middle East, and Africa), Countries: Bulgaria, Germany, Italy, Lithuania, Malaysia, Poland, Spain, United Kingdom; APAC (Asia-Pacific), Countries: Australia, India, Malaysia, Singapore, Taiwan. CI, confidence interval. ^a^ was considered statistically significant.
